# Study of post-partum mental disorders: case of the Hospital of Mental Health and Psychiatric Diseases of the Chu Mohammed-VI of Oujda

**DOI:** 10.1192/j.eurpsy.2025.1473

**Published:** 2025-08-26

**Authors:** S. Essadki, R. Hammouch, A. Berka, Y. El Aissaoui, R. Boughoufa

**Affiliations:** 1 Higher Institute of Nursing professions and Health Techniques; 2 THE HOSPITAL OF MENTAL HEALTH AND PSYCHIATRIC DISEASES OF THE CHU MOHHAMED-VI, Oujda; 3 Laboratory of Life and Health Sciences, Faculty of Medicine of Tangier, Tangier, Morocco

## Abstract

**Introduction:**

The birth of a child is a joyous event that can be accompanied by major challenges for women’s mental health. It is marked by physical, hormonal, psychological, family and social upheavals. It can also be associated with an increased risk of incidence or decompensation of psychiatric disorders.

**Objectives:**

The aim of this study is to describe the trends and characteristics of post-partum mental disorders in the Orientale region of Morocco.

**Methods:**

36 files of patients hospitalised or having consulted for a post-partum psychological disorder at the Mental Health and Psychiatric Diseases Hospital of the Mohammed-VI University Hospital Centre in Oujda were examined using a data collection form.

**Results:**

The main disorders identified were post-partum depression and puerperal psychosis, with rates of 62% and 38%, respectively. 32% of them have attempted suicide, 14% have suicidal thoughts, 32% have committed infanticide, and 21% have rejected their baby. With regard to pregnancy and childbirth, a third of the women had a caesarean section, with primiparous women predominating. 57% of the women described their childbirth experience as long and painful. Similarly, the use of addictive substances was mentioned in 44% of cases.

**Conclusions:**

It is true to say that measures need to be put in place to improve conditions for women and to raise awareness among those around them of the need for early detection of these disorders. These proposals underline the importance of taking concrete steps to support the women concerned and promote their mental well-being.

**Disclosure of Interest:**

None Declared

